# Nutritional quality of heat-sensitive food materials in intermittent microwave convective drying

**DOI:** 10.29219/fnr.v62.1292

**Published:** 2018-10-10

**Authors:** Nghia Duc Pham, W. Martens, M.A. Karim, M.U.H. Joardder

**Affiliations:** 1 Science and Engineering Faculty, Queensland University of Technology 2 George street, Brisbane, QLD 4001, Australia; 2 Engineering Faculty, Vietnam National University of Agriculture, Vietnam

**Keywords:** intermittent microwave convective drying, Kiwifruit, microstructure, ascorbic acid, Polyphenol, colour analysis, nutritional quality, drying characteristics

## Abstract

**Background:**

The retention of health promoting components in nutrient-rich dried food is significantly affected by the dehydration method. Theoretical and experimental investigations reported in the literature have demonstrated that intermittent microwave convective drying (IMCD) can effectively improve the drying performance. However, the impact of this advanced drying method on the quality food has not been adequately investigated.

**Design:**

A programmable NN-SD691S Panasonic inverter microwave oven (1100 W, 2450 MHz) was employed for the experiments. The microwave power level was set at 100 W and ran for 20 seconds at different power ratios and the constant hot air conditions was set to a temperature of 60°C and 0.86 m/s air velocity.

**Objective:**

In this study, natural bioactive compounds (ascorbic acid and total polyphenol), water activity, colour and microstructure modifications which can occur in IMCD were investigated, taking kiwifruit as a sample.

**Results and Discussion:**

The microwave (MW) power ratio (PR) had significant impact on different quality attributes of dried samples. The results demonstrate that applying optimum level MW power and intermittency could be an appropriate strategy to significantly improve the preservation of nutrient contents, microstructure and colour of the dried sample. The IMCD at PR 1:4 was found to be the ideal drying condition with the highest ascorbic acid retention (3.944 mg/g DM), lowest colour change (ΔERGB = 43.86) and a porous microstructure. However, the total polyphenol content was better maintained (3.701 mg GAE/g DM) at higher microwave density (PR 1:3). All samples attained a desirable level of water activity which is unsusceptible for microorganism growth and reproduction.

**Conclusion:**

Overall, IMCD significantly improved the drying performance and product quality compared to traditional convective drying.

**Nomenclature**

**Table:** 

IMCD	Intermittent microwave convective drying
*h*	Hue angle
M	Moisture content of the material (g/g DM)
M_e_	Equilibrium moisture content (g/g DM)
M_o_	Initial moisture content (g/g DM)
ΔE_RGB_	Total colour difference
AAC	Ascorbic acid content (mg/ g DM)
GAE	Gallic acid equivalents (mg/ g DM)
PR	Power ratio
Rpm	Round per minute
a_w_	Water activity
DM	Dry mass (g)
t_on_	Microwave on time
t_off_	Microwave off time
RGB	Red – green – blue
TPC	Total polyphenol content

One of the major processes in food industries is drying. Drying adds value to a product, reduces transportation and storage costs, increases a product shelf life and has the ability to improve the bio-accessibility and bio-availability of health-promoting compounds in food (1). However, the quality of heat-sensitive plant-based food rapidly deteriorates during conventional drying, which results in considerable destruction of bioactive components and development of undesirable colour, flavour, texture and microbiological spoilage (2). In order to shorten the lengthy processing time in traditional convective drying, high-temperature drying medium can be used. However, these types of drying methods generally result in quality deterioration, in particular, a high reduction in their nutritional value (3, 4). To overcome this issue, an integrated convective and microwave (MW) drying method has been proposed. This hybrid drying method has a potential to improve energy efficiency while maintaining desirable product quality by utilising the advantages of each drying method (2, 5). This hybrid drying method can significantly reduce the processing time due to rapid volumetric heating caused by MW radiation. However, continuously applying the MW and convective heating can result in higher and localised temperatures. These elevated temperatures can cause significant quality deterioration in thermo-labile foods like fruits and vegetables (6). Due to the continuous supply of heat, the atoms and molecules of bioactive components’ movement are accelerated, which increases the frequency of collisions until the products reach the sufficient energy to start the chemical reaction. The rate of biological reaction approximately doubles for every 10°C increase in temperature (7–9). Prolonged exposure of samples to a drying environment can facilitate the degradative reactions (i.e. vitamin C, polyphenol oxidation, protein hydrolysis and beta-carotene isomerisation), which eventually reduces the health-promoting benefits and antioxidant activity of bioactive ingredients in plant-based food materials. One such drying method which has received attention recently is intermittent microwave convective drying (IMCD). Applying MW radiation intermittently during convective food drying has shown huge potential to reduce browning effects, reduce mitigation of hydro-thermo-mechanical stresses generated inside the sample, and minimise the chemical reactions that lead to health-beneficial component losses and adverse physical quality modifications (10–13). The results show that this style of drying is a promising alternative for drying of thermo-labile plant-based food materials. This advanced hybrid drying method has two sections within the drying cycle, the active drying period and the tempering period (2, 14). During the active drying period (when MW is on), moisture from the food surface is evaporated and carried away, while in the tempering period (when MW is off) the temperature and moisture are redistributed from the high- to low-concentration regions. Due to the moisture gradient, moisture still migrates from the interior region to the surface in the tempering period of the drying cycle. This allows the high drying rate to be maintained for the next active cycle. This cycle of cooling and rewetting the sample during the tempering period prevents samples from overheating and quality deterioration. Overheating can also be avoided by controlling the MW supply to the sample allowing the energy to be wisely ultilised, and therefore, the sample is heated within the safe region ensuring the food quality. Hence, IMCD has the potential to provide an optimal drying process by overcoming the issues associated with continuous hot air and/or continuous MW drying (15–17).

Currently, despite the considerable potential of IMCD, the impact of IMCD on food quality has not been extensively examined (2, 18, 19), particularly no study has investigated the effect of IMCD on the nutritional quality attributes of kiwifruit. Kiwifruit (*Actinidia chinensis Planch*) is an attractive, valuable and nutrient-dense fruit with inviting green/yellow flesh colour, distinctive flavour, taste and many health beneficial ingredients. It is an abundant source of chlorophyll, actinidin, total polyphenol, vitamin E and vitamin C with a high level of antioxidant capacity while containing no fat or cholesterol (20). Because of its abundant health benefits, a growing interest is being observed in the impact of thermal dehydration on the properties of these compounds and their relation to other quality characteristics. Kiwifruit is one of the highly perishable foods with seasonal availability; many preservation methods have been attempted to prolong their storage life and make this healthy product commercially available to consumers (21–25). IMCD drying could be one potential dehydration method able to extend kiwifruits shelf life and add value by efficiently reducing moisture content, hence inhibiting microbial growth and deteriorative chemical and enzymatic reactions (26).

Kiwifruit has been investigated for convective drying by several researchers (21, 27–30). Similarly, changes in colour (25, 31, 32) and physical properties (33, 34) of hot air convective dried kiwifruit have been published. The influence of conventional drying conditions on bioactive compounds in fresh and dried kiwifruit has also been examined by Tian et al. (22), Orikasa et al. (23) and Kaya et al. (24). However, no study exits of the impact of IMCD conditions on the drying characteristics and quality attributes of kiwifruit. It is especially challenging to produce high-quality dried kiwifruit as the fresh kiwi contains high amount of thermal-sensitive elements while intensive heating treatments generally have detrimental effects on the bioactive compounds, colour, water activity and microstructure of the product. Therefore, a comprehensive research is required to obtain an appropriate drying method to ensure the quality of nutrition-rich fruits like kiwifruit.

In this context, the primary aim of this study is to investigate the quality characteristic change of kiwifruits under different IMCD conditions. The retention of AAC and total polyphenol content, water activity, colour degradation and microstructure modification were investigated under different IMCD PRs, taking convective air-dried sample as a reference.

## Materials and methods

### Sample preparation for drying

Fresh green Nutri kiwifruits were used for the IMCD experiments. They were carefully chosen to be identical in shape, size, firmness and ripeness. The fresh samples were stored at 4 ± 1°C before experiments. The kiwifruits taken from the laboratory fridge were washed with distilled water to remove residue and dirt and then allowed to reach room temperature before conducting each drying experiment. The initial moisture content of the fresh kiwi slices was estimated to be approximately 83.4 ± 0.03 (% w.b.). The kiwifruit skin was peeled, and then slices of 50 mm diameter and 7mm thickness were made by cutting perpendicularly to the main core fruit axis using a food slicer. One slice of kiwifruit was dried in each IMCD condition and three replicates were carried out.

### Drying equipment and experimental procedure

For IMCD experiment, a programmable NN-SD691S Panasonic inverter MW oven with a maximum energy output of 1,100 W (2,450 MHz) was employed for the experiments. The inverter MW oven provides constantly true power transmission at the setting values. The MW power level was set at 100 W and was turned on for 20 sec at different PRs, and the hot air drying time was in accordant with PR during the MW time off. The hot air condition was set to a temperature of 60°C and 0.86 m/s air velocity. The kiwifruit slice was placed in the centre of the MW cavity, for an even absorption of MW energy. The moisture loss was recorded at regular intervals at the end of power-off times by placing the sample slice on the digital balance. Once the kiwifruit had reached a moisture content of approximately 19.5 ± 0.4 (% w.b.), the drying process was stopped. Sample temperature was regularly checked during the drying process by aFlir E5 thermal imaging camera to record the maximum sample temperature.

The PR (PR = t_on_ : t_off_) was set at four different modes: 1:1, 1:2, 1:3 and 1:4. Here t_on_ was the MW on time in seconds and t_off_ was the MW off time in seconds.

An independent hot air convective drying experiment was also conducted to compare with the results from IMCD, which had the same experimental conditions as in IMCD method.

The decrease in the moisture ratio (MR) with the drying time was used to examine the experimental data. The MR denotes the remaining moisture in the kiwifruit samples in relation to the initial moisture content, which can be calculated by Equation (1).

$$MR=\frac{M-M_{e}}{M_{o}-M_{e}}$$ (1)

Where *M* is the moisture content of the material on a dry basis, *M*_e_ is the equilibrium moisture content, and *M*_o_ is the initial moisture content. For an extended period, the *M*_e_ becomes very insignificant and considerably small as compared to *M* and *M*_o_, and therefore, Equation (1) can also be written as *MR = M/M_o_* (35, 36).

### Water activity measurement

The water activity (a_w_) was measured using an Aqualab water activity meter (Decagon Devices, Pawkit, USA) in stable laboratory condition at 24 ± 0.3°C. The dried samples of different drying conditions were kept in a desiccant chamber until the sample temperature reach the room temperature before placing in the sample cup of the water activity meter. The water activity measurements of dried product were performed in triplicate.

### HPLC analysis of AAC content

About 4 g of the fresh sample or 1 g of dried sample was homogenised for 1 min at maximum speed in a UltraTurrax T25 homogeniser in 25 ml of metaphosphoric acid buffer (3% metaphosphoric acid, 1 mM Na_2_EDTA) under low light condition, purging with nitrogen to prevent the oxidation process. The homogenised samples were vortexed, then filtered through a Whatman no. 3 filter paper and diluted again to 25 mL of the extractant solution and centrifuged at 10.000 g for 15 min at ambient temperature.

AAC contents were determined based on the HPLC method proposed by Asami et al. (37) with some modifications. The analysis was carried out using an Agilent HPLC 1100, G1311A dual pump, G4225A 1260 HiP Degasser, equipped with a G1315B DAD absorbance detector. The reverse-phase separation was obtained using a Waters Symmetry C18 column (4.6 × 250 mm, 5 μm). The isocratic mobile phase was HPLC graded water brought to pH 3.0 by metaphosphoric acid. The flow rate was 0.5 mL/min; injection volume was 20 μL, and the applied wavelength was 254 nm. Supernatants of the extracted samples were filtered through a 0.22 μm Acrodisc syringe Nylon membrane filter before injection. The retention time of AAC peak was archived at 2.6 min. A 5-point standard curve was established to calculate the ascorbic content of samples. All samples were tested in triplicate.

### Total polyphenol extraction and measurement

The amount of total polyphenols in the samples was determined by Folin–Ciocalteau method (38) with some modification. About 4 g of the fresh sample or about 1 g of dried sample was homogenised in 25 mL of a mixture of acetone and distilled water (70:30 v/v) for 1 min at maximum speed (25,000 rpm) in a UltraTurrax T25 homogeniser. The homogenised samples were shaken and then allowed to settle for 1 h at ambient temperature. Extracts were centrifuged at 10,000 g for 15 min at 20^o^C. The supernatant added with 5 mL extractant was filtered through a Whatman no. 3 filter paper. Extraction processes were repeated three times to get reliable data, and the extracts were diluted 10 times with distilled water. Then, 1 ml of the diluted extract solution was mixed with 5 ml of Folin–Ciocalteau reagent (10%) and 4 ml of 7.5% Na_2_CO_3_ solution. After 30 min of incubation in a water bath at 37°C, the absorbance was measured against water at the highest absorption wavelength with kiwifruit extraction solution, 760 nm (Cary50 UV Spectrophotometer). A standard curve with gallic acid standard solutions was established. The amount of total phenolic content (TPC) was in milligrams of gallic acid equivalents/gram (GAE) on the dry mass basis.

### Microstructure

A scanning electron microscope (Model Mira 3 Tescan, Kohoutovice, ČeskáRepublika) was used to examine the microstructure of the dried kiwifruit slices. The scanning electron microscopy (SEM) analysis was used in order to determine the degree of change/damage of the kiwi cells caused by the drying process. The fresh/dried kiwifruit samples were cut into cubes of 5–8 mm^3^ by shaft razor blade, placed on a SEMstub by carbon strip, then sputter coated with 10 nm gold before observation (Leica EM SCD005 sputter coater). Fresh samples were scanned under low vacuum conditions (40 Pa), LVSTD detector, at an accelerating voltage of 10 kV; while dried samples were scanned under high vacuum mode, 10kV voltage, SE detector.

### Image acquisition and colour analysis

Digital images were obtained using 8.0 megapixels Samsung camera. These images were stored in the bitmap graphic format. ImageJ software was employed to analyse digital images in the RGB (Red–green–blue) colour. RGB-triplets for every pixel in the image represent the intensities of RGB colours in the range 0–255. Before processing of the sample images, the preprocessing was carried out based on a method proposed by Sharifian et al. (39) to avoid the non-uniform light distribution in the background and to remove surrounding noises. Colour measurement was performed in triplicate in each drying condition to determine hue angle and colour change (6). The hue angle *h*^o^ is defined as:

$$h^{o}=tan^{-1}\left(\frac{\sqrt{3}\left(G-B\right)}{2R-G-B}\right)$$ (2)

Therefore,

$$tanh^{o}=\left(\frac{\sqrt{3}\left(G-B\right)}{2R-G-B}\right)$$ (3)

Colour changes were defined as in Equation (4):

$$\Delta E_{RGB}=\sqrt{{\left(\Delta R\right)}^{2}+{\left(\Delta G\right)}^{2}+{\left(\Delta B\right)}^{2}}$$ (4)

Where ΔE_RGB_ represents the total colour changes of IMCD dried kiwifruit slices compared to the fresh sample.

### Statistical analysis

The investigated characteristics were independently performed in triplicate. The mean data were analysed by analysis of variance (ANOVA) using SAS (version 9.1; SAS Institute Inc., Cary, NC, USA). Least significance difference (LSD) tests were employed to determine the difference of means, and statistical significance with a confidence interval of 95% (*p* ≤ 0.05).

## Results and discussion

### Drying process

**Fig. 1:**
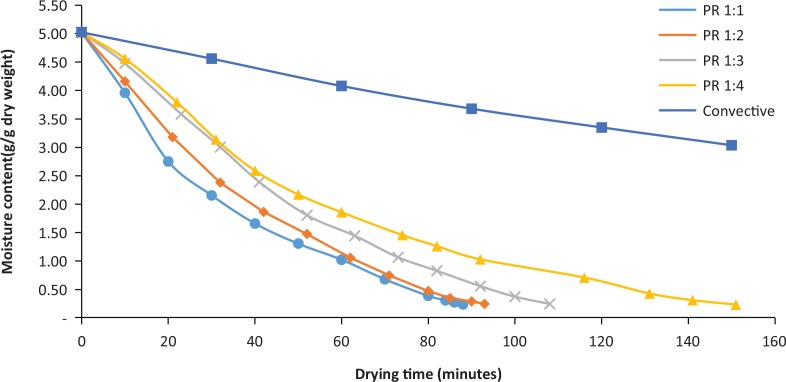
Drying curves at different drying conditions.

Experimental drying curves for different PRs of IMCD and the reference convective drying curve are presented in Fig. 1. It can be seen that the processing times required to attain the same final moisture content were different at different drying conditions (PRs). The drying time required to reach the moisture content of 19.5 ± 0.4 (% w.b.) from the fresh condition of 83.4 ± 0.03 (% w.b.) at PR 1:1, 1:2, 1:3 and 1:4 were 88 min, 93 min, 108 min and 151 min, respectively, whereas convective drying took 528 min to bring the sample to the same moisture content. While in all IMCD settings, the moisture content reached <20% within 150 min, the convective sample still remained at a high moisture content, approximately 75 (% w.b) or 3.04 (g water/g dry mass). The results demonstrate that IMCD can significantly reduce the drying time compared to convective drying.

The drying rate was significantly high at the beginning of the IMCD process as the initial available moisture content in the samples was high. The drying rate was then gradually reduced towards the end of the drying process. It can be concluded that in the early drying stage, the MW energy was mainly absorbed by water near the sample surface, the free and loosely bound water of the sample, which was easily removed (40).

It is clear from Fig. 1 that a higher PR led to higher drying rate during the drying process, resulting in a shorter drying time. Because of the volumetric heating characteristics of MW, the MW energy directly penetrated inside the sample and excited water inside the sample. At the same time, the sample was gradually heated from the outer surface of the sample by the hot air (Kumar et al. 2016). The moisture outward flux was eventually increased, and drying rate increased considerably. However, there was an insignificant difference in the drying times between drying PR 1:1 and 1:2, especially in the final drying period (when the moisture content was <1 g/g dry basis), indicating that determining right PR in IMCD is critical to increase the drying rate and achieve shorter drying times.

### Water activity

Another essential quality indicator measured in this study was water activity, which allowed determining the product stability and safety (41). Water activity characteristics determine many chemical or enzyme reaction and biochemical processes, which are important for the control of food product safety and quality. The results of water activity measurement in fresh and dried kiwifruits are presented in Table 1. The fresh material was characterised by the average water activity of 0.97 ± 0.012. According to Sablani et al. (42), the growth of microorganisms, the microbiological stability of food materials, depends on the value of water activity. Moreover, a change in water activity affects the relative speed of chemical, enzymatic and biological reaction (42, 43). This can be seen in Table 1; the water activity of all dried samples was less than 0.6, which means that the growth of bacteria, yeasts and moulds will be inhibited and the degradation of chemical reactions will be minimised in all the drying conditions considered. However, the highest a_w_ value was obtained in convective drying (0.57 ± 0.011) and the lowest a_w_ value was observed in IMCD at PR 1:1 (0.34 ± 0.004), which was about 40% lower compared to convective drying. In drying pumpkin slices, Junqueira et al. (44) found similar results that MW drying provided lower water activity in the samples than other methods. Water activity describes the bound of moisture to a food’s structure and it is related to the energy required to remove moisture from the sample. It also takes part in chemical/biochemical reactions and growth of microbials. Therefore, the results demonstrated that the intensity of MW heating takes a significant part in the rate of bound water removal from the sample, which is considered as the most time-consuming process and ineffective energy use in conventional hot air drying. The result suggests that MW effectively reduced the sample water activity to the optimum stability level. Attaining low water activity at desired moisture level helped to extend product shelf life while maintaining its expected quality characteristics.

**Table 1:** Water activity of samples under different drying conditions

Samples	Water activity
Fresh sample	0.97 ± 0.012
Convective drying sample	0.57^a^ ± 0.011
IMCD PR 1:1 sample	0.34^d^ ± 0.004
IMCD PR 1:2 sample	0.41^c^ ± 0.005
IMCD PR 1:3 sample	0.43^c^ ± 0.011
IMCD PR 1:4 sample	0.52^b^ ± 0.005

Different superscript letters (a, b, c, d) indicate significant differences (*p* ≤ 0.05) among the samples under different drying conditions. Identical superscript letters indicate no significant difference.

### Ascorbic acid

**Fig. 2:**
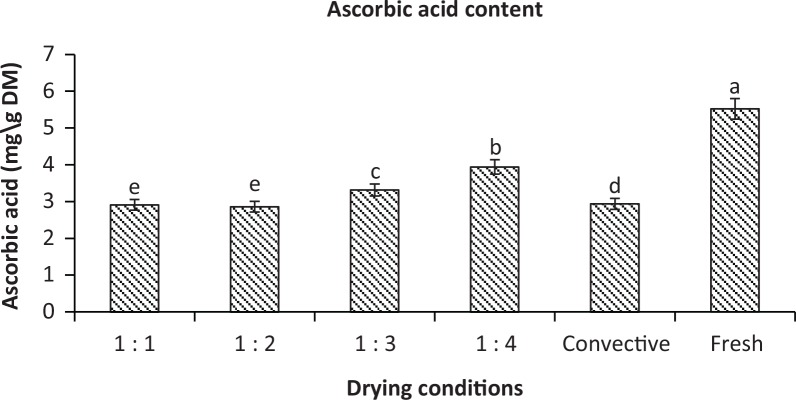
Ascorbic acid content of fresh and dried sample slices under different PRs 1:1, 1:2, 1:3, 1:4 and convective drying. Different letters (a, b, c, d) indicate significant differences (*p* ≤ 0.05) among the samples under different drying conditions. Identical letters indicate no significant difference.

AAC is one of the most thermo-labile bioactive compounds in plant-based foods. Therefore, the thermal drying processes should be carried out in a way that ensures the highest preservation of AAC in the dried food products. The effects of IMCD at different PRs and convective drying on the retention of AAC in dehydrated kiwifruit are shown in Fig. 2. It can be seen that drying conditions greatly affected the retention of AAC and its content decreased in all drying conditions in comparison with the fresh samples. This phenomenon could be due to the thermal destruction during drying and the exposure of the samples to the drying medium. The degradative chemical reactions of natural bioactive compounds are catalysed by heat and ascorbate oxidase enzyme released from disrupted cell membranes during the drying process. The average total AAC found in fresh kiwifruit was 91.64 mg/100 g fresh sample (5.520 mg/g DM), which is consistent with those reported in several varieties and genotypes of fresh green kiwi: 59.65 mg/100 g (45), 65–120 mg/100 g (46), and 117.65 mg/100 g (24). Because AAC is a heat-sensitive nutrient, higher degradation of AAC was observed in convective drying and IMCD at higher PR (e.g. 1:1 and 1:2) and the retention of AAC was approximately 52%. The temperatures of samples were found fluctuated during each IMCD cycle, which was at peak point at the end of the MV heating period and lowest point at the end of the tempering period. The maximum temperatures recorded were 70°C, 76°C, 81°C and 85°C at PR 1:4, PR 1:3, PR 1:2 and PR 1:1, respectively. The higher frequency of MV radiation (PR) increased the sample temperature during IMCD, which generally degrades the heat-sensitive bioactive compounds within the sample. This result affirms the thermal sensitivity of AAC at intensive heating conditions and lengthy drying process of the samples. The results of this study show that the highest retention of AAC (*p* ≤ 0.05) can be obtained at drying conditions of PR 1:4 (3.994 mg/g DM) which is a pretty mild condition and relatively short total drying time, retaining approximately 71.5% of AAC from the fresh sample, whereas samples dried with hot air drying at 60°C retained only 53.2% of AAC (2.938 mg/g DM), which was slightly higher than the retention of AAC at PR 1:1 and PR 1:2. It is also noted that the total drying time is significantly longer in convective drying.

### Total polyphenols content

**Fig. 3:**
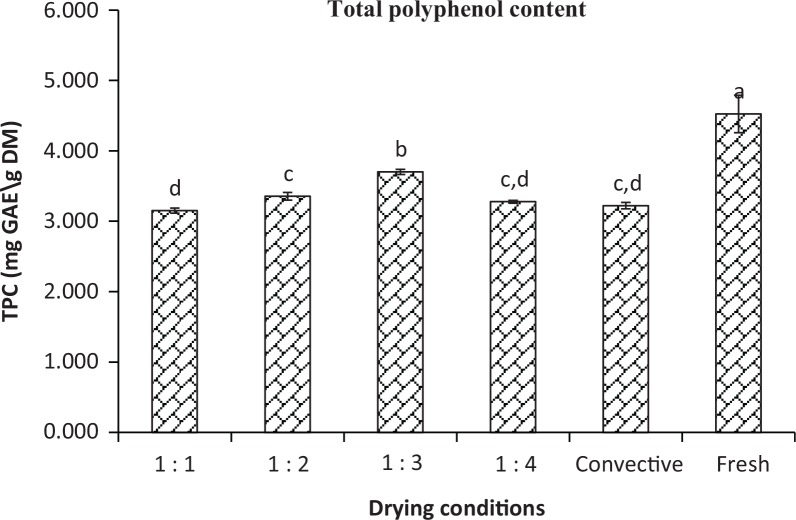
Total polyphenol content of fresh and dried sample slices under different PRs 1:1, 1:2, 1:3, 1:4, and convective drying. Different letters (a, b, c, d) indicate significant differences (*p* ≤ 0.05) among the samples under different drying conditions. Identical letters indicate no significant difference.

The retention of total polyphenol content of the fresh and dried kiwi slices is shown in Fig. 3. The average concentration of the total polyphenol in fresh kiwifruits was 4.523 mg GAE/g DM. At the end of the drying process, the highest loss of 30.4% of the TPC had occurred at PR 1:1 (3.150 mg GAE /g DM), followed by 22.7 and 23.5% loss in PR 1:2 and PR 1:4 samples, respectively, while the loss of TPC was lowest (14%) at PR 1:3. A significant difference (*p* ≤ 0.05) in the TPC between PR 1:4 and PR 1:3 was observed. In spite of the fact that the drying condition in PR 1:3 was more heat-intensive than PR 1:4, it is interesting to observe the highest polyphenol retention at PR 1:3 (3.701 mg GAE/g DM) . The above-mentioned results also demonstrated that the level of TPC retention in IMCD is higher than that of AAC. It can be concluded that TPC is less heat sensitive than AAC. Also, the MW radiation at PR 1:3 might be sufficient to release the bound polyphenols by breaking the cellular food matrix for better extraction in chemical analysis. The discharge of the oxidative and hydrolytic enzymes from the collapsed food cells can decompose long-chain polyphenols into simple phenolic compounds. Also, newly formed phenolic compounds were the result of the complex heat catalysed chemical reaction of the released enzymes and bioactive compounds. In addition, more phenolic products can be produced as the result of Maillard reaction under PR 1:3. In contrast, thermal destruction by intensive MW heating in PR 1:2 diminished polyphenols and longer drying time in PR 1:4 and convective drying gained sufficient time to facilitate oxidative deterioration of polyphenols (47–49). The obtained results also highlighted the advantages of combining hot air and intermittent MV to enhance extraction efficiency of the bioactive compounds due to its combined effect on the cell membrane. Moreover, the elevated temperature induced by MW radiation at suitable PR can reduce the degradation of polyphenols by inactivating the polyphenol oxidase, lipoxygenase and peroxidase enzymes released from the collapsed tissue (50). Many published articles (51–53) reported that MW drying retains high total polyphenol. On the contrary, an insignificant difference (*p* > 0.05) in the TPC was observed between drying mode PR 1:4 and convective drying, with the retention of approximately 75% (3.277 mg GAE/g DM). Overall, compared to fresh samples, all the dried samples retained high level of phenolic contents. The lowest phenolic retention was approximately a third from fresh sample observed in IMCD at PR 1:1 as well as in the case of convective drying.

### Microstructure

**Fig. 4:**
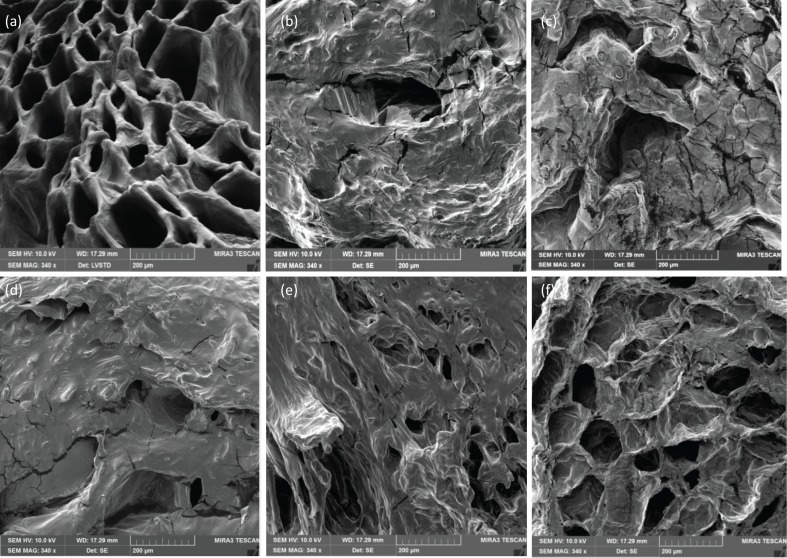
Microstructure of sample slice in fresh condition and dried state under different drying conditions. (a) Fresh samples, (b) PR 1:1 sample, (c) PR 1:2 sample, (d) convective sample, (e) PR 1:3 sample, and (f) PR 1:4 sample.

As MW penetrating deep into the samples causes volumetric heating and rapid moisture evaporation, it is suspected that significant microstructural changes may take place during IMCD. However, no investigation on microstructural changes in IMCD can be found in the literature. Illustration of the microstructure of fresh and dried samples derived from different drying conditions has been presented in Fig. 4. Prolonged external heating in convective drying (60°C) causes case hardening and collapses as shown in Fig. 4d. A limited number of pores and cell opening can be observed. The hot air drying induced slow water migration with high turgor reduction, structural shrinkage and collapse, whereas hot air drying coupled with periodic MW heating provided a more porous structure compared to the purely convective drying method as depicted in Fig. 4.

The MW reduces the drying time due to rapid moisture evaporation from inside the material. However, cell collapse can also be observed due to overheated regions resulted from uneven heating during MW heating at higher PR. This incident is observed from Fig. 4b and c that represents the cracked and collapsed microstructure of sample dried at PR 1:1 and PR 1:2.

It is also noted that there is swelling, loss of stability and disappearance of many cell walls of kiwifruit structure in the convective dried sample as well as IMCD samples at high PRs. It can be explained that the kiwifruit cells are composed of a small amount of cellulose, and a high amount of pectic polysaccharides (54), which were degraded significantly during severe thermal stresses in hot air convective and higher PRs in IMCD. Therefore, the prolonged heating time in continuous hot air drying and/or intensive MW heating (PR 1:1 and PR 1:2) resulted in the adverse microstructural changes in kiwifruit. The IMCD drying condition PR 1:3 and PR 1:4 maintained better kiwifruit microstructure. IMCD at PR 1:4 obtained a porous structure resembling the structure of the fresh sample, less shrinkage and collapses (Fig. 4f) compared to the other drying modes. Tian et al. (22) reported similar results for MW-assisted vacuum drying which indicated that convective sample had a less porous structure with severe cell disruption, while MW vacuum drying maintained open pore structure with good appearance. The modification of sample microstructure might also affect the other sensor properties, for example, hardness and crispiness. Highly porous structure obtained at PR 1:3 and PR 1:4 tends to produce crunchy and crispy products, while dense and collapsed structure in dried products at PR 1:1 and PR 1:2 usually results in chewy and hard products.

### Colour degradation

During drying, sample colour can be considerably affected by pigment degradation (55, 56), enzymatic activity (57), and Maillard nonenzymatic browning reaction (58). The changes in porosity and surface texture also vary the reflectance of light on food surface, which ultimately affects viewers’ colour perception (5, 59–61).

The visual appearance, average RGB values along with total colour changes altered by different drying conditions are presented in Table 1. It is clearly seen that higher PR caused more colour degradation in the sample.

Similar to the change of nutrient content, sample colour was also negatively influenced by higher PR because of the accelerated colour degradation reaction occurred within the sample due to higher MW irradiation. The application of MW heating brought about the rapid colour change of the samples, caused by intense thermal effect and browning reaction during drying. It is also noted that extended drying time in convective drying significantly degraded the colour of the dried product. From Table 2, it can be stated that a higher PR led to higher colour changes in the sample. However, the maximum colour change (ΔE_RGB_= 69.56) (*p* ≤ 0.05) was found in the case of hot air drying, while minimum colour changes (*p* ≤ 0.05) took place in PR 1:2, 1:3 and 1:4 IMCD mode.

**Table 2:** RGB values and total colour changes of samples under different drying conditions.

Factor	Sample PR 1:1	Sample PR 1:2	Sample PR 1:3	Sample PR 1:4	Sample convective	Fresh sample
	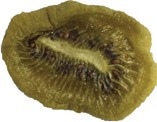	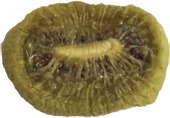	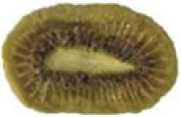	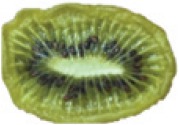	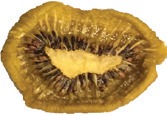	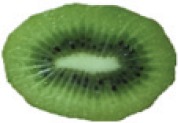
**Red**	119.97 ± 21.88	127.42 ± 20.28	119.25 ± 24.51	146.67 ± 20.29	166.45 ± 32.35	105.45 ± 16.00
**Green**	103.22 ± 21.62	109.77 ± 21.53	102.26 ± 25.39	159.17 ± 22.79	123.25 ± 30.87	144.61 ± 17.73
**Blue**	41.03 ± 16.02	51.66 ± 16.79	49.51 ± 18.32	71.76 ± 25.17	42.52 ± 23.56	68.22 ± 15.72
**ΔE_RGB_**	51.60 ± 2.58^b^	44.40 ± 2.04^c^	48.31 ± 2.32^b,c^	43.86 ± 2.06^c^	69.55 ± 3.37^a^	-

Different superscript letters (a, b, c, d) indicate significant differences (*p* ≤ 0.05) among the samples under different drying conditions. Identical superscript letters indicate no significant difference.

**Fig. 5:**
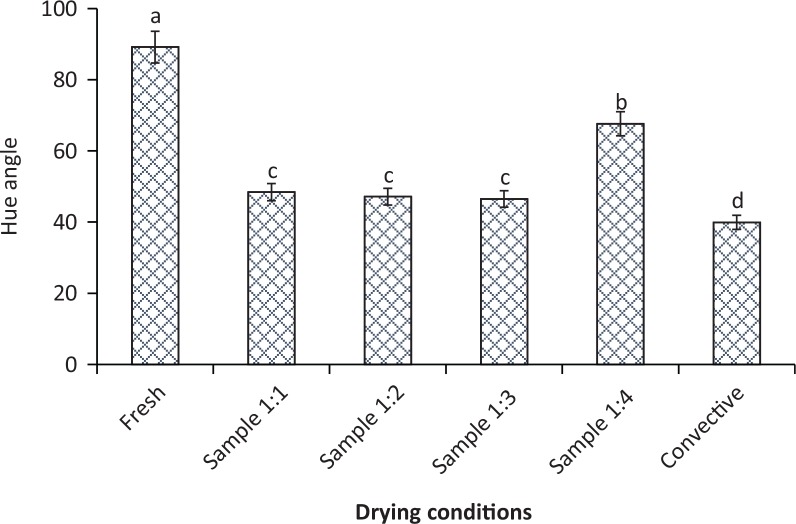
Average hue angle of the dried sample under different PRs 1:1, 1:2, 1:3, 1:4, and convective drying. Different letters (a, b, c, d) indicate significant differences (*p* ≤ 0.05) among the samples under different drying conditions. Identical superscript letters above the bars indicate no significant difference.

In addition to colour changes, the hue angle of the dried samples in convective drying, as well as IMCD, was considerably different from the fresh sample as shown in Fig. 5. In light of this finding, it is clear that the hue angle (values from 40 to 68) implies high colour changes at IMCD PR 1:1, 1:2 and 1:3, but there was an insignificant difference among these IMCD conditions (*p* > 0.05). However, the colour of the product under these conditions was still better than in the case of convective drying (*p* ≤ 0.05). Hue angle of the sample dried in IMCD with the lowest PR (PR 1:4) resulted in the closest hue angle to the fresh sample (*p* ≤ 0.05). Reducing the PR further may result in less degradation of colour (closer to the hue angle of the fresh sample). However, it will increase the total drying time, which might also negatively affect colour as extended time exposure to drying environment facilitated browning reaction as in the case of convective drying. Therefore, PR 1:4 can be considered the best operating condition for the best colour, AAC retention and achievement of shorter drying time.

The product qualities, as described in the above sections, decrease with the increase of PR due to higher temperature induced by intensive MW heating. Although some reports in the literature claimed that decreasing PR might provide better food quality, it would prolong the drying time and increase the overall energy consumption (3, 62, 63). Moreover, as demonstrated above, prolonged drying time can cause undesirable effects on the quality of the samples. Therefore, an appropriate/optimum PR should be determined for the optimisation of drying time and food quality. In the experiments of IMCD, the tempering period supported the redistribution of the temperature and moisture, especially at lower PR as it allowed sufficient time to even out the temperature and moisture difference in the sample (64). Therefore, the product quality was better maintained in IMCD at lower PRs.

Considering the above discussion, it can be concluded that better appearance and quality of dried food can be achieved by appropriately setting the MW power-on/off time in IMCD drying.

## Conclusion

Effect of the operating variable (e.g. PR) of IMCD on the drying process and product quality were investigated in this study. In the IMCD experiments, the drying time was significantly reduced in comparison with conventional hot air drying process. The water activity of all dried samples was attained at a safe level for extended storage. Thus, the microbial growth has been inhibited, and degradation of chemical reactions has been retarded. In addition, water activity decreased with the increase in PR of IMCD resulting in safer products with a longer shelf life. Higher PR and/or extended exposure time to the drying environment resulted in a greater degradation of the total polyphenol and AAC values. It was observed that the PR was a profound factor in the IMCD on the health-promoting compounds (AAC and total polyphenols) and colour as well as the microstructure. The best drying conditions in this study were identified at PR 1:4 (MW 20 sec on/80 sec off at 60°C hot air) in terms of colour and AAC retention. However, it should be noted that IMCD at PR 1:3 retained the best total polyphenol. It was found that the IMCD method was substantially more efficient than convective drying as it significantly decreased the drying time and enhanced the product nutrient and colour quality with a porous microstructure. In addition, the highly porous microstructure of IMCD samples suggests better texture compared to hot air dried product. However, further lowering PR might not guarantee better quality product (e.g. PR 1:4 attain lower total polyphenol content than at PR 1:3) and efficient drying performance due to prolonged exposure of the products to the drying environment. Therefore, the PR should be carefully chosen based on product quality, MW power and drying performance to achieve optimal drying conditions.
